# Reporting Standards for Bayesian Network Modelling

**DOI:** 10.3390/e27010069

**Published:** 2025-01-15

**Authors:** Martine J. Barons, Anca M. Hanea, Steven Mascaro, Owen Woodberry

**Affiliations:** 1 Department of Statistics, University of Warwick, Coventry CV4 7AL, UK; 2 Centre of Excellence for Biosecurity Risk Analysis, University of Melbourne, Parkville, VIC 3052, Australia; anca.hanea@unimelb.edu.au; 3 Bayesian Intelligence, Upwey, VIC 3158, Australia; steven.mascaro@bayesian-intelligence.com (S.M.); owen.woodberry@bayesian-intelligence.com (O.W.); 4 Faculty of Information Technology, Monash University, Clayton, VIC 3800, Australia

**Keywords:** Bayesian networks, reproducibility, decision support, systematic review, policy, reporting standards

## Abstract

Reproducibility is a key measure of the veracity of a modelling result or finding. In other research areas, notably in medicine, reproducibility is supported by mandating the inclusion of an agreed set of details into every research publication, facilitating systematic reviews, transparency and reproducibility. Governments and international organisations are increasingly turning to modelling approaches in the development and decision-making for policy and have begun asking questions about accountability in model-based decision making. The ethical issues of relying on modelling that is biased, poorly constructed, constrained by heroic assumptions and not reproducible are multiplied when such models are used to underpin decisions impacting human and planetary well-being. Bayesian Network modelling is used in policy development and decision support across a wide range of domains. In light of the recent trend for governments and other organisations to demand accountability and transparency, we have compiled and tested a reporting checklist for Bayesian Network modelling which will bring the desirable level of transparency and reproducibility to enable models to support decision making and allow the robust comparison and combination of models. The use of this checklist would support the ethical use of Bayesian network modelling for impactful decision making and research.

## 1. Introduction

Reproducibility [1,2,3] is a key requirement for assessing the veracity of a modelling result or finding and for judging the reliability and value of a model. In this paper, we primarily use the term ‘reproducible’, sometimes interchangeably with ‘replicable’ and ‘repeatable’, as per Fidler and Wilcox [1]. Governments and international organisations are increasingly turning to modelling approaches in the development and decision making for policy. This trend has a long history, see, e.g., Kraemer and King [4], and is ongoing. On the dimensions.ai research analysis platform, publications containing ‘government policy modelling’ as a subset of those containing just ‘government policy’ has grown from 24% in 2015 to 36% in 2024.

Bayesian Networks (BNs) are a class of graphical models capable of efficiently representing the probabilistic dependencies amongst a set of variables [5,6,7]. They consist of a directed acyclic graph (DAG) coupled with conditional probability functions (often conditional probability tables or CPTs) that define the probabilistic relationships between variables. Due to their ability to represent causal and statistical relationships directly and efficiently, they have found use across a range of disciplines with differing research cultures and statistical capabilities. They have natural integrations with decision theory, providing probabilities conditioned on evidence and actions that can be combined with utilities, either directly within the models or subsequently by the human decision makers. BNs support decision making in environmental [8], management [9], agricultural [10], industrial [11,12], medical [13] and many other domains.

The ethical issues of relying on modelling that is biased, poorly constructed, constrained by heroic assumptions and not reproducible for policies affecting many lives are clear [14]. The ethical concerns with BNs are the same concerns relating to any statistical methods, and they may arise due to selection and other biases, problems with internal validity, misreporting, manipulation of results, professionalism, transparency [15] and little to no transportability to other settings [16]. A key concern with highly multivariate models (such as many BNs) is their complexity, making them more difficult to understand [17] and in some cases having them be the equivalent of opaque black boxes; when data are used as (or processed to produce) evidence for a conclusion, it is reasonable to expect that the connection between the data and the conclusion should be accessible [18] regardless of the algorithm or method used. Reporting sufficient details on the model and the modelling process is the primary mitigation against this issue, and this also works to combat the other problems described. The failure to report sufficient detail to reproduce models or outcomes undermines the ability of the user to have confidence in the results and their robustness. The European Commission, in partnership with the EC Competence Centre on Modelling, recently issued guidance on the questions to be asked when presented with the use of simulation models in policymaking [19], a supplement to Data Science guide for Society [20], which equips policymakers with the right questions to ensure accountability in data-led decisions. The questions cover data, its provenance and availability; the assumptions built into the modelling; the availability of model inputs; code and development; the suitability, performance and scrutiny of the model (including by peer review); and the communication of policy-relevant model outputs, including the uncertainties and the limitations with discussion of their impact on results.

In order to ascertain whether a study has yielded fruitful research for the good of society its development, assumptions, modelling choices, results and conclusions need to be supplemented by sufficient detail to allow others to replicate the study and uncover any shortcomings. The medical literature is well ahead of many other disciplines, with major journals and funding bodies strongly encouraging or mandating the inclusion of EQUATOR [21] (Enhancing the QUAlity and Transparency Of health Research) checklists with the submission of manuscripts. EQUATOR brings together a range of checklists tailored for different study types as follows: CONSORT for randomised Trials, STROBE for observational studies, PRISMA for systematic reviews, etc.

The reproducibility crisis in economics and psychology has begun to be addressed through the development of reporting checklists, being inspired by the medical sciences [22] and encoding the recommendations and guidelines developed for using and reporting statistical methods and good practice in those disciplines. In 2015, a study found that of 100 psychology research findings, 39 could be replicated and 24 attempts at replication produced ‘moderately similar’ results to the original, casting doubts on the veracity of the remaining 37 [23]. One approach to mitigate this problem is open science, where researchers share their methods, data, computer code and results in central repositories [24]. Preregistering a study, using large samples and sharing research materials are associated with high replicability [25]. Considering social sciences more widely, an open science approach is considered to be a safeguard against failure to reproduce results and misconduct [26]. Government and international organisations increasingly apply behavioural science research to direct human decisions, amplifying the critical nature of rigour in social science research.

In addition to policy making and ethical considerations, the accurate, transparent and complete reporting of studies is crucial, as published articles are a primary source of information and knowledge for the research community ([22], p. 29). If insufficient detail is reported, researchers cannot critically evaluate the scientific methods and merits of a study. The usefulness of studies for research synthesis, meta-analysis and the links between study characteristics and replication success is diminished with insufficient reporting.

Often, the goal of a replication study is to reproduce a particular result under the same experimental conditions as used in the original study. This is called ‘outcome reproducible’ by Gundersen [2] and is often appropriate for domains such as medicine and psychology, where the interventions and outcomes are well-defined (including observational studies such as those based on target trial emulation [27]). However, this is uncommon for most kinds of BN study. In particular, the ‘result’ is often a complex mechanistic or statistical multivariate model of some system, with potentially thousands of parameters, while these parameters can be independently meaningful and reproduced, and they may sometimes be the target for reproduction (for example, in a small causal BN). For most BN studies, we would typically be most interested in reproducing the development process and analysis that produced the BN and any key qualitative results derived from it.

This is what Gundersen [2] calls ‘analysis reproducible’. Currently, there is no clear guidance for those wishing to achieve this kind of transparency and reproducibility. In particular, there are no reporting guidelines that provide a minimum list of information needed to ensure a manuscript or report can be understood by a reader, reproduced by a researcher, used by a decision maker and included in a systematic review. In this paper, we have sought to construct a question-based checklist to support the best practice reporting for BN models.

## 2. Materials and Methods

The authors of this paper, all experts in BNs, specified an initial set of eight reportable features of a BN and its development process which were considered essential for reproducing a study. The initial list of reportable features consisted of the following:

1. **Template/bespoke**: Whether the model is based on a template or whether it has been developed from scratch for the problem.
2. **Variable choice**: The variables included in the model.
3. **State space/discretisation**: The state spaces of the variables (for discrete variables) or the discretisation used (for continuous variables).
4. **Model definition**: The definition of the model structure, variables and relationships.
5. **Conceptual model**: If a conceptual model was developed prior to BN development, the description of this conceptual model. In this context we think of a conceptual model as a high-level mind-map of the key components of the problem to be modelled and their relationships.
6. **Edges**: The directed edges or arrows in the model.
7. **Parameters**: The parameters needed for whatever relationship holds between parents and children (e.g., the conditional probability table/function, correlations, regression coefficients).
8. **Validation/Verification**: Any tests of the model to ensure that it is (a) internally consistent and correct and (b) meets the modelling purpose.

The authors then reviewed their own and others’ published work on BNs to see how these features were reported, as well as to identify any further features from the publications that appeared necessary for reproducibility. The 15 papers used for this extraction were [28,29,30,31,32,33,34,35,36,37,38,39,40,41,42]. This led to a revision of the list of reportable features, involving the refinement of the list to minimise the overlap between features (ideally, to achieve mutual exclusivity), while also elaborating on the properties of the features that would be reported on, including, *who* was involved, *how* the feature was developed and *where* the feature was defined and described. For example, one of the reportable features at this stage was the BN structure, and the three properties of this feature were (1) who was involved in the development of the structure, (2) what method was used to develop the structure and (3) where the definition of the structure was described. Other features (such as template/bespoke) consisted of only one property. This resulted in a list of 16 reportable features, which were used as the basis of a draft questionnaire. The draft questionnaire was implemented as an interactive tool and was then developed iteratively.

As the development of the questionnaire via the online tool proceeded, the authors opted to create a longer reporting form than originally intended, allowing for better guidance, the broader coverage of modelling tasks and better reproducibility. This included the development of a detailed help text that accompanied each of the items, automatically made visible as the reporter works through the items. The authors drafted and included the help text prior to circulating the questionnaire for feedback, allowing questionnaire reviewers (i.e., the researchers who reviewed the questionnaire tool during the feedback process) to provide feedback on both the questions and the associated help text, giving them the best chance of understanding the intent of the questions.

The authors chose to divide the questions into four key sections as follows: model information, structure, parameters and evaluation. The sections were chosen to align with common checkpoints during model development, acknowledging in the tool (specifically, the help text) that model development is iterative. Several questions contained repeated patterns of sub-questions, and these patterns were formalised so as to be consistent across sections. The resulting survey design and structure is described fully as part of the results (in Section 3.1).

### Feedback and Evaluation

To recruit reviewers for the questionnaire, the draft questionnaire tool was circulated to academic and professional colleagues who have worked with BNs (all with multiple years of experience) as well as board members of the Bayesian Network Modelling Association (BNMA). BNMA is an association based primarily in Australasia that aims to promote the understanding, use and research into BNs, and it has held conferences since 2009. Board members generally have a strong background and experience with BNs. There were 11 reviewers who responded over the course of the evaluation, 3 of whom were BNMA board members. Reviewers were asked to fill in the questionnaire based on one of their pre-existing studies and were asked for feedback on what was missing that would be needed to allow for its reproduction. They were also asked to assess if the questionnaire was clear and easy to answer, as well as the usefulness, correctness and clarity of the help text. Reviewers were explicitly told that the usability, length, burden and format of the questionnaire and website were not a focus of the review.

There were two rounds of feedback. In the first round, reviewers were asked to examine the tool (without filling in the questions) and provide unstructured (free text) feedback via separate comments in a document or via email. This feedback was used to revise the questionnaire tool, leading to the addition of an opening explanatory statement, revisions to the help text, expansion of conditional questions by default (for the feedback process) and additional opportunities for section-specific feedback throughout the questionnaire. In the second round, reviewers were encouraged to submit information based on a BN model from one of their own projects; further feedback was also collected during this process directly via the tool itself. All feedback was anonymised, collated and summarised by topic, and this summary is presented in Section 3.2.

After the final feedback was collected, the authors checked the tool for outstanding issues. Subsequently, three authors employed the tool to review 5 BN application papers each (15 in total). This took approximately 20–30 min per paper to complete. These consisted of 10 entirely new papers [43,44,45,46,47,48,49,50,51,52], and 5 from the original 15 papers used to design the original questionnaire. This provided an evaluation of the applicability of the questionnaire and a preliminary indication of the reporting frequency for each item in the community at present.

## 3. Results

The final questionnaire developed by the authors is described in Section 3.1, including an overview of the reportable items and the reasoning for the inclusion or omission of items and concepts. The results of the questionnaire evaluations, both via the feedback process and via the retrospective evaluation of past papers, are discussed in subsequent sections (Section 3.2 and Section 3.3, respectively).

### 3.1. The Questionnaire

#### 3.1.1. Model Information

In order to reproduce or assist the reproduction of a BN study, several key pieces of general information or metadata are needed, and the authors collected this information under the *Model Information* section. Perhaps the single most important piece of BN metadata included was the model purpose. Other key information included the following: what tools were used to develop the BN, the (online) location where the BN is stored or defined and who had input into the design and creation of the model. The *Model Information* section (Table 1) was designed to be short and the authors omitted less relevant metadata. Some of the notable omissions are described here.

The authors recognised that BNs are applied to many different kinds of problems and make use of diverse BN features; hence, they do not always take the same form. There are discrete and continuous models, qualitative and quantitative models, latent variable models; some BNs are intended to be read causally, while others imply only probabilistic dependencies. The authors also considered that there are still further variations that extend or use BNs in more distantly related ways. In epidemiology, (causal) directed acyclic graphs (DAGs) have been separated from BNs and are used to guide what has come to be called causal inference, the statistical analysis of individual causal relationships. Hierarchical Bayesian models generalise further from traditional discrete BNs, frequently containing more complex graphical structures that cannot be represented with DAGs alone (for example, by using plate models). These models allow hierarchical hyperparameters to be specified over lower level probability distributions. Structural equation models are also closely related, sufficiently so that structural causal models have been proposed (by Judea Pearl [53]) as a direct functional analogue of causal Bayesian networks.

While the authors regarded *all of these* as belonging to the same class of model representation, all addressable by the questionnaire, the authors chose to tailor the questionnaire to more traditional BN development approaches. Hence, there were no questions included for statistical analysis plans (relevant for causal inference), hyperparameters and hierarchical structure design and the use of simulation techniques (relevant for hierarchical Bayesian models), and none for intervenable variables and checks on whether noise terms are independent (relevant for structural causal models). Despite omitting these items, the authors did not intent to imply that good practice in reporting is satisfied without them where relevant and did not rule out expanding the scope of the reporting standards to include them in the future.

Even within the realm of traditional BN models, there is a diversity of techniques that would require a more comprehensive interactive questionnaire. Some of the most common include dynamic Bayesian networks (DBNs), decision networks (DNs) and object-oriented Bayesian networks (OOBNs). The authors observed that reporting on DBNs for reproducibility would require specifying additional details such as stationarity, within and between time slice structures; timing information; expected roll out sizes; burn-in requirements; and expected convergence behaviour, as well as needing to deal with DBN elements that are part of a larger, non-dynamic BN. Tailored questions for these and other techniques were omitted in favour of keeping the questionnaire concise and focused.

The authors also discussed that many BNs are also programmatically generated—the most common examples are DBNs where full BNs are often generated from a BN fragment representing one timeslice along with the number of timeslices desired. For such BNs, it is difficult to give a clear answer to questions around node, structure and parameter definitions because they depend on how the BN generation is done. The authors initially included a question on whether the BN was programmatically generated, but this led to complications and the questionnaire now assumes that models are either developed statically or otherwise a particular generated instance has been chosen for reporting, with the details, if important, left for an associated paper or report to cover.

#### 3.1.2. Structure

The BN structure includes the definition of the variables and the topological relationships between those variables. The authors recognised that the BN structure is tightly bound to the language and relevant concepts of the problem; hence, it would be critical for the reporter to provide clear definitions and interpretations for reproducibility (Table 2). Hence the emphasis on these topics in this section.

The authors recognised two key approaches to defining the structure of a BN, which could be used alone or in combination. The BN structure can be derived from data, in which case details of the learning algorithm would be required for reproduction. Alternatively, the BN structure can be derived from expert judgement, either using a formal protocol [54], an adaptation of an existing model [55] or facilitated discussion. The key details of these approaches are covered by the items relating to knowledge sources (see Section 3.1.5 and Table 5).

##### Node Dictionaries

Language differences between expert domains and signature pedagogies [56] can cause disagreements between experts in eliciting the BN structure. Carefully facilitated discussion can often reveal that the same concepts are being described in different language. Sometimes the opposite happens; experts use the same or similar language and only after some time realise that the same word has different meanings, or different nuance of meaning in different expert domains or pedagogies. With skilful facilitation, a common language can be agreed on for the purpose of a project. For this reason, as well as for reproducibility, a node dictionary which defines the meaning and measurement of each node (including, where appropriate, cut points selected in making a continuous node into a discrete one) are included in the checklist.

##### Causal Structure

Whether a BN’s structure is intended to have a causal interpretation, a statistical interpretation, or a combination of the two (when only parts of the structure can be interpreted casually), affects many aspects of the BN development process. Causal structures can be used directly to support decision making (with tools such as Pearl’s do calculus and utility theory) as well as causal effect estimations (with the do calculus again, along with other tools such as inverse probability weighting and g-estimation), and they provide causal explanations for the processes they represent. There are standard tools (built into most BN software packages) that can perform these tasks. By contrast, these standardised tools would give invalid results for non-causal models (in which only statistical relationships are claimed)—further work would be needed to process the inputs and outputs for a non-causal model to achieve the same goals. Hence, it is important that users know when a BN is intended to be interpreted causally, permitting these additional tools to be validly applied.

If a BN is to be interpreted causally, additional information is required for reproducibility. In particular, domain experts with the appropriate causal knowledge need to be involved, either in the design or validation (or both) of the causal structures. For BNs that have been derived from automated structure learning, the learning methods should be validated causal discovery algorithms (i.e., algorithms that have been validated for their ability to recover causal structures); in the cases where the causal discovery methods cannot recover the causal structure, an explanation of how expert causal knowledge has been used to decide the structure is necessary. The authors have included this advice in the help text for the item on causal structure.

#### 3.1.3. Parameters

Information about parameters most often depends on the BN structure and rarely the other way around (with the BN structure depending on the parameters, like in the case of structural zeroes). Hence, the Parameters section follows the Structure section in the questionnaire order. The authors identified that key information about the parameters needed for the reproduction of a study (Table 3) include basic details such as the parameter types and values, the way in which parameter values have been discretized or defined and methods used to estimate the parameters, whether from experts or data.

Parameters, just like the structure, can be derived from (the same) multiple knowledge sources. Despite sharing the same types of knowledge sources, the authors recognised that it is very common for different types of sources to be used for structure and parameters. An example of one common split is to have experts help define the structure, but use data for the parameters. Hence, the questionnaire allows knowledge sources to be specified separately for both.

#### 3.1.4. Evaluation

Evaluation can come at any stage of the development process, but only after some aspect of the BN (such as variables, dependencies or parameters) has been specified. Hence, the authors chose to make evaluation the final section, while acknowledging that it may take place at almost any stage during development. While details of the evaluation (Table 4) are not needed to reproduce the creation of the BN, they are needed to reproduce claims that may have been made about the BN, which are equally important.

Validation, verification and even evaluation are often used interchangeably, and sometimes inconsistently, across many different domains. The original meanings are, for validation, to show that something is logically valid and potentially logically sound (that the argument is correct and the conclusion true if the premises are true), and for verification, to show that something is true (e.g., provide evidence of the truth of a conclusion by providing evidence of the truth of the premises). However, in software engineering, and many other disciplines, validation has come to take on the more specific meaning of “have we built the right product”, i.e., “have we given the right answer”. Verification has come to equate “have we built the product right” with “are we using a process that will yield the right answer”—neither of which correspond to the original meanings of the two words.

As a consequence, and to avoid inconsistent responses, the authors ask these questions together. The help text provides advice on what these terms mean, leaning in favour of the meanings used in software engineering, to be as consistent as possible with what is likely to be the norm amongst BN, simulation and computational modellers generally.

#### 3.1.5. Common Features

##### Knowledge Sources

The source from which knowledge is derived is relevant to all aspects of a BN, and is critical to understanding how confident one can be in the BN. Knowledge sources are often inter-related—experts are a knowledge source that derive their knowledge from other sources such as the research literature; literature is often based on the knowledge derived from data analysis, and so forth. Nevertheless, the way in which information is collected for a particular study typically depends on the source, as do the implications of the collected information. Hence, we ask what sources have been used divided by common types, along with follow up questions that are tailored to each source (Table 5 and Table 6). For example, if the user indicates experts as a source, the questionnaire asks how many and what kinds of expertise; meanwhile, if they indicate data was used as a source, we ask what tools were used for processing and learning and whether the data was used for development, validation or for both.

The authors also recognised that expertise is, by its nature, often unique and can make reproduction much more difficult. However, the chances of successful reproduction and useful understanding can be improved by a reporter providing sufficient detail on the experts contributing to the structure development and the methods used to elicit those contributions. The authors noted that, in their experience, the nature of expert judgement is such that the same number of experts with the same balance of expertise frequently produce similar (potentially equivalent) descriptions of the system being modelled, even though there may be differences in the language and finer details.

The details for the structure and parameter knowledge sources were sufficiently similar so that items were presented in the same way (Table 5), while the items for evaluation knowledge sources were presented somewhat differently (Table 6) but consisted of the very same knowledge source types.

##### Question Format

Initially, the authors designed many of the questions to be multiple choice. The user would be able to select from a list of the most common techniques, approaches or formats, or they would be able to provide a custom description if none matched. After initial mock-ups were created, it became clear that it would be difficult to identify and develop robust lists of common techniques just with reference to the literature, and so the lists were extracted and incorporated into a separate help system.

##### Help System

Having identified the elements considered essential for reporting a BN so that it could be reproduced, the authors drew on their experience with building BN models (across multiple domains and with partners from different academic and application domains) to inform the design of the help system for the resulting questionnaire.

The help system was designed to be an integral part of the questionnaire, providing essential details for some questions where the language and concepts may not not be considered standard. It also described in brief some of the most common practices, and the authors intended it to encourage broader standardisation in the use of terms and language for BN model development. A key goal in outlining these common practices was to provide assistance to novice model developers. Hence, the aim was to provide sufficiently broad and appropriate coverage of these techniques without it being overwhelming for the novice modelling audience.

### 3.2. The Testing

The questionnaire was implemented on a dedicated platform and shared with reviewers not involved in the initial stages of development. Reviewers were selected based on their background as theoreticians and/or practitioners heavily involved in BN modelling. They were instructed to provide specific feedback on four main aspects as follows:

1. What additional questions would be needed to reproduce the recorded model/study?
2. Are the questions clear and easy to answer for the reported model/study?
3. How useful and complete is the help text associated with each question?
4. Do the sections follow an appropriate and structured order?

Feedback on usability, length and format was not requested and reviewers were advised not to focus on these issues. However, some aspects of usability did arise in answering the above questions, with many commenting on potential misunderstandings due to the way the questions were sequenced or due to unfamiliar terminology. The latter will be addressed further (as part of addressing the second question), as for the former, no specific comments (that would also address the fourth question) were made.

Eleven reviewers provided feedback across the two feedback rounds, most of which was positive and encouraging. Suggested additional questions covered the following: a detailed workflow describing the development process for the BN (including number of workshops, meetings and surveys run; number of modellers and experts involved, etc.); model calibration (in addition to verification and validation); more options for types of connections and types of variables (non-causal, functional, degenerate distributions); and more details about the expert judgement protocols (especially if an unstructured process was used, and also to be able to assess the burden on experts). Reviewers also suggested extra questions surrounding data use, in terms of sources; general availability; and sample sizes.

In terms of the clarity of questions, most feedback related to terminology and the use of terms in different contexts (e.g., “*Parameters* is a term that has many meanings”, “Evaluation/sensitivity is also used differently in different contexts”, nodes vs. variables). It was noted that because the models can combine causal modelling, simulation and statistical models, the meaning of an analyst/modeller can be understood differently.

Several questions were identified as sensitive (rather than not easy to answer), specifically those requesting identification of the problem owner(s) and stakeholder(s). These sensitivities arise, for example, when these roles are not officially defined, or when they come with a pressure to break assumptions around privacy/confidentiality (by reporting the names of additional people that provided input to the model’s development).

Other aspects of usefulness, completeness and appropriateness (covered by the last two questions in the required feedback) did not receive many comments from the reviewers, but the few who did comment were extremely positive.

Maybe the most important, general criticism, which occurred in the first round of feedback, was that the objective of the questionnaire was not clear (e.g., “Often it was not clear to me what you were asking, so I just guessed […]. I never guessed that it was reproducibility you were after.”, “[…] the objective of your work on this (not really described)”). This may also be a consequence of the chosen format of the questionnaire (which did not provide an integrated explanatory statement and did not provide specific feedback questions). In addition, when evaluating the existing feedback, it became clear that because of the format (of both the questionnaire and the feedback process), parts of the questionnaire were not addressed or accessed by some of the participants. This was partly because some of the questions were conditional, and some of the reviewers did not trigger the reveal of the conditional questions. A lack of clarity in the succession of questions led to misunderstandings about parts of the detailed answered we sought.

The objective and format of the questionnaire was refined prior to the second round of feedback and the responses subsequent to the revision contained no doubts about the motivation. However, new potentially missing information was identified as follows: what output would the tool provide to researchers reporting on their BNs, where would their reported information go and how might the questionnaire be useful for researchers? A new iteration will add the ability to generate a report from the information provided (as had originally been planned), and the tool’s guidance will be revised to further clarify the use of the generated report, with the potential to submit this report together with a publication.

Other comments addressed the need for a more concrete characterisation of the BN, in terms of direct questions about the total number of nodes/variables, or the total number of arcs. These questions had been omitted to keep the questionnaire short and were not considered critical for reproduction (since the information could be extracted from other details reported); however, a future version of the tool will likely allow this information to be entered, or potentially automatically extracted.

### 3.3. Current Reporting Standards

There were 15 papers used to evaluate the questionnaire as follows: 5 from the original 15 papers used to design the questionnaire, plus another 10 not used in the development. The extra 10 papers used for evaluation were [43,44,45,46,47,48,49,50,51,52]. A summary of these evaluations can be found in Table 7. A detailed list of the evaluations can be found in Appendix A. Since the reporting tool was applied only approximately, in order to produce a general sense of item reporting frequencies, individual papers have been anonymised to protect authors from small mistakes in the evaluation process, potentially implying the unwarranted criticism of the papers.

Some of the key results are summarised below.

#### 3.3.1. Model Information

Most of the time, the BN tools and roles were clearly stated. A model purpose was always provided, but it was well described in only about half of the cases. It was rare for the paper to provide an online version of the BN, highlighting a significant impediment to reproducibility.

#### 3.3.2. Structure

A node dictionary was provided about 70% of the time. Only half of the BNs were identified explicitly as causal or non-causal (and most but not all of these were causal). Most of the time, the BN Structure was given as a figure in the paper. In cases where it was not given, it was typically because it was generated.

About two thirds of the BN structures were built using expert elicitation. Of these, 70% gave details about the processes and protocols used during expert elicitation and only half of those provided good detail about those processes. The number of experts and their details were only provided around half the time. The literature sources used to build BN structure were typically noted, but there very rarely were citations included in the node dictionary. About half of the BN structures were built using data, and in all cases, the tools and processes were described, and typically very well. However, none of the papers for which citations to data sources would be relevant to structure data learning included citations in the node dictionary. This may be due to datasets being used for the full BN rather than for parts of the BN.

#### 3.3.3. Parameters

Almost all of the papers provided enough information on the variable types used. Most of them included node states or discretisations in the node dictionary, as well as providing the BN parameters in some form.

Less than half of the BNs used expert elicitation for parameters, and of those, only half described the processes and protocols used. Again, the number of experts and their details were only provided around half the time. Most of the time literature sources used to parameterise BNs were described, and citations were occasionally included in the node dictionary (but more frequently than for structure). While two thirds of BN structures were built with expert elicitation, about two thirds of the BN parameters were learnt from data. In almost all cases, the tools and processes used for learning were described. Only half the time were the priors used described, which is low considering that *all* learning begins with some prior, even if uniform or uninformative. Some of the papers included citations of the datasets used in the node dictionary.

#### 3.3.4. Evaluation

Nearly 60% of the papers described the BN validation and verification processes, yet roughly 80% of the papers described some sensitivity analysis process.

Almost none of the papers used expert evaluation, or when they did, it was not described adequately. About two thirds of the papers used statistical evaluation, and 60% of those adequately described how the data were used. Most of the papers described clearly the separation between datasets used for building and evaluating the BN. Roughly 70% also provided citations for the evaluation data.

## 4. Discussion

Bayesian networks are valuable and widespread models, especially in development and decision making for policy. Many aspects of the BN models are well reported, including the model purpose, node dictionary and the process and protocols used in the expert elicitation of the BN structure. However, priors, data sources relevant to structure learning and the number and details of experts used in elicitations are often not well reported. The questionnaire in this study was designed to be widely applicable in BN modelling work. It takes approximately 20 min to complete for a research report or a scientific publication. (An online version of the questionnaire is made available by the BNMA at https://reporting.bnma.co/, accessed on 22 December 2024). Feedback from reviewers along with our own evaluations against existing studies in the literature provide evidence that the questionnaire achieves this wide applicability, but stronger evidence requires the broader use of the tool amongst BN developers, while we expect to develop these reporting guidelines as more information emerges from its real world use, (as is common with such guidelines. See, for example, CONSORT’s evolution [57]), we believe the evaluation we have done so far supports releasing the questionnaire and guidelines to the wider BN community today. The evaluation suggests the guidelines are beneficial. Moreover, releasing them now would allow us to shape these guidelines together with the BN community into the future, basing this on its application to real world studies.

Feedback from the reviewers of the checklist showed that most of the important developmental stages of BN modelling were covered. However misunderstandings most evident in the first round of feedback, arising from the formatting and differences in terminology, highlight the importance of ensuring the tool is usable and can be understood by researchers from diverse backgrounds. These issues were addressed successfully for the second round of feedback; however, as the tool is released to a much broader audience, issues like these will almost certainly need to be revisited. Despite this, many of the reviewers applauded the initiative of standardising BN reporting, even via the first round of feedback.

In reaching out to sections of the BN community to co-produce this checklist, we anticipate that it will urge its widespread use. Engaging with the community has helped us to identify the kinds of items that are important to the community, align the language and approach and better convey the use and value of standardised reporting. Unlike in medicine and epidemiology, standardised reporting is not very common in AI and knowledge representation. The BN community, however, partly due to the level of expert involvement in many projects and the unique difficulties that this poses, recognises the need for better approaches to communicating project methods and results to support reproducibility.

In addition, the existence of the checklist and guidelines may support those new to BN modelling. This includes early career researchers who are entering BN modelling as a field of study, whose professional development can be supported by considering transparency and reproducibility in the formative stages of their careers. It may introduce them to techniques and tools of which they may not yet be aware, and provide them with an initial glimpse of what they can do and where that best applies, often within the context of their own project.

The checklist may also support researchers whose primary expertise is in other fields but who wish to harness the power of BN modelling for their research. The questionnaire can act as a quick-start guide to an established researcher from another domain, providing them with an understanding of the essential ingredients for any BN development project and an overview of some recognised best practices.

The checklist may support those who are reviewing BN modelling for transparency and reproducibility to support the research community. For many reviews, the guidelines can act as a quick summary of whether a submission meets common BN community standards. In addition, it has been the unfortunate experience of the authors on more than one occasion that reviewers outside the BN community are unaware of standard good practices within the BN community. The guidelines can help these reviewers improve the quality of their critiques both directly and indirectly, if responses to the questionnaire are submitted explicitly alongside a paper with a pointer to the guidelines.

In a similar vein, the guidelines may help journals advise authors on the appropriate level of detail for their BN modelling—editors and reviewers can point authors directly to this checklist if they feel that a submission falls short of the details needed for understanding and reproduction. It can also help commissioners of research and those applying for funding to ensure that relevant details are reported to allow the work to have an ongoing benefit beyond the lifetime of the specific project.

As noted, the guidelines do not cover many kinds of common extensions to BNs, as well as closely related modelling techniques that one might think should be included. BNs are quite complex and flexible, so we intentionally began with the most tractable forms. However, this is a limitation that we hope to address in future versions of the guidelines, particularly based on the information we gain from further real world use of the current guidelines. We expect this will allow us to expand the guidelines relatively easily to support structural causal models and dynamic BNs, as well as the (non-quantitative) DAGs used for causal inference. More significant work is likely needed to make the reporting of other types of models (such as decision networks, object-oriented BNs or other template-based BNs and Bayesian hierarchical models) generally useful.

Ideally, an expanded version of the reporting standards would be integrated with open access systems for archiving and sharing BNs. A key advantage of BNs that is yet to be exploited fully by the BN community is that the representations and knowledge they contain are reusable. One of the impediments to reusing BNs has been a lack of standardisation in representational approaches. While this is unlikely to be resolved in the short-term, better reporting can help researchers identify relevant research and BN models much more effectively, and it may stimulate the sharing of knowledge resources. There are several public repositories of BN models online; however, we intend to first work on an integration with the BN repository provided by the BNMA (https://bnma.co/bnrepo, accessed on 22 December 2024).

Finally, standardised reporting provides much stronger transparency into the modelling process, providing decision makers with better, comparative information that can be used to assess the suitability of BN models for decision making. This makes it easier for all involved to identify what contribution the BN modelling has made to individual decisions and management processes, and where processes and practices can be improved. This can be of great value for small modelling projects that affect only a few, as well as for larger projects that affect many.

## Figures and Tables

**Table 1:** List of items in the Model Information section.

Item	Entry Type
Title	Text
Author (s)	Text
Problem owner (s)	Text
Analyst/Modeller (s)	Text
Other key stakeholders	Text
Model purpose	Long text
Citation (if applicable)	Text
BN available online	Yes/No
URL	Text (Conditional)
BN tools	Text
Other comments	Long text

**Table 2:** List of items in the Structure section.

Item	Entry Type
Have you included a node dictionary?	Yes/No
Where is the node dictionary (or alternative) reported?	Text
Is the structure causal?	Yes/Partially/No
Where is the structure defined?	Text
Select all applicable knowledge sources	Knowledge source (see Table 5)

**Table 3:** List of items in the Parameters section.

Item	Entry Type
Select variable types used in the model.	Discrete/Continuous/Mixed
Have you included state/discretisation definitions in node dictionary?	Yes/No/Elsewhere
Where?	Text (Conditional)
Are the parameters described?	Yes/No
Where?	Text (Conditional)
Select all applicable knowledge sources	Knowledge source (see Table 5)

**Table 4:** List of items in the Evaluation section.

Item	Entry Type
What validation/verification processes were used?	Long text
What sensitivity analysis processes were used?	Long text
Where is the evaluation reported?	Text
Select all applicable knowledge sources.	Knowledge source (see Table 6)

**Table 5:** Knowledge source questions shared by the Structure and Parameters sections.

Item	Entry Type
Select all applicable knowledge sources	Any of: Analyst/Modeller, Problem Owner, Domain Expert(s), Literature, Data, Other please specify
**Expert Elicitation Details** (Conditional section)	
Where is the elicitation process described?	Text
Which elicitation processes/protocols were used?	Long text
How many experts were involved?	Text
Provide details on the expert fields of expertise	Long text
Other comments	Long text
**Literature Details** (Conditional section)	
Where are the literature sources described?	Text
Are literature sources cited in the node dictionary?	Yes/No/Elsewhere
Where?	Text (Conditional)
Other comments	Long text
**Data Learning Details** (Conditional section)	
Where is the data learning process described?	Text
What data learning tools/processes were used?	Long text
Are data sources cited in the node dictionary?	Yes/No/Elsewhere
Where?	Text (Conditional)
Other comments	Long text

**Table 6:** Knowledge source questions specific to evaluation.

Item	Entry Type
Select all applicable knowledge sources	Any of: Analyst/Modeller, Problem Owner, Domain Expert(s), Literature, Data, Other please specify
**Expert Evaluation Details** (Conditional section)	
Where is the expert validation reported?	Text
How were experts used?	Long text
Were the experts different from model development?	Yes/No
How many experts were involved?	Text
Provide details on the expert fields of expertise.	Long text
Other comments	Long text
**Data Evaluation Details** (Conditional section)	
Where is the data validation reported?	Text
How were data used?	Long text
Were the data different from model development?	Yes/No
Are data sources cited in the node dictionary?	Yes/No/Elsewhere
Where?	Text (Conditional)
Other comments	Long text

**Table 7:** The frequency of item reporting for the items in the Reporting Standards BN questionnaire.

	YY	Y	N	NA	Reported %
**Model Information**					
Roles	2	10	3	0	80%
Model purpose	7	8	0	0	100%
BN online	3	0	12	0	20%
BN tools	7	5	3	0	80%
**Structure**					
Node dictionary	3	7	4	1	71%
Causal	3	5	7	0	53%
Structure definition	10	3	2	0	87%
**Structure Expert Elicitation**					
Process/protocols	4	3	3	5	70%
Num experts	2	2	6	5	40%
Expert details	2	3	5	5	50%
**Structure Literature**					
Sources described	2	7	2	4	82%
Citations in node dictionary	0	1	9	5	10%
**Structure Data Learning**					
Tools/processes	5	2	0	8	100%
Citations in node dictionary	0	0	5	10	0%
**Parameters**					
Variable types	3	11	1	0	93%
States/discretisation in node dictionary	4	7	3	1	79%
Parameters described	5	7	3	0	80%
**Parameter Expert Elicitation**					
Process/protocols	3	0	3	9	50%
Num experts	1	2	3	9	50%
Expert details	0	4	2	9	67%
**Parameter Literature**					
Sources described	2	5	1	7	88%
Citations in node dictionary	0	2	4	9	33%
**Parameter Data Learning**					
Tools/processes	5	5	1	4	91%
Priors	0	4	5	6	44%
Citations node dictionary	3	0	5	7	38%
**Evaluation**					
Validation/verification processes	3	5	6	1	57%
Sensitivity processes	4	7	3	1	79%
**Evaluation Expert**					
How were experts used?	1	0	5	9	17%
Different from build	0	0	0	15	
Num experts	0	0	1	14	0%
Expert details	0	0	1	14	0%
**Evaluation Data**					
How were data used?	3	3	4	5	60%
Different from build	2	4	1	8	86%
Citations (where)	2	2	2	9	67%

## Data Availability

Data are contained within the article.

## Appendix A

### Appendix A.1. Help Text for Model Information Page

- **Model information (Default):** Provide high level details about your model, such as title and authors, on this page.
- **Title:** Give a formal title or name of the BN, report, document, or project.
- **Author(s):** Identify the person or group of people responsible for creating or authoring the BN, report, document, or project.
- **Problem owner(s):** Identify the individual or individuals responsible for addressing and managing the problem the model is designed to address.
- **Analyst/Modeller(s):** Identify the individual or individuals responsible for conducting data analysis or modelling tasks. For a BN project, this might be the expert in modelling technology.
- **Other key stakeholders:** Identify individuals, groups or organizations with a vested interest in the subject matter or outcome related to this BN, report, document or project.
- **Model purpose:** Provide a clear and concise explanation of the intended purpose of the model being referred to. Specify why the model was created, what it is designed to accomplish or how it contributes to the overall project or analysis.
- **Citation (if applicable):** Provide a citation for your BN, report, document or project.
- **BN available online** Indicate whether the model is available online or accessible through a specific platform. For example, the Bayesian Network Modelling Association (BNMA) has a repository where you can upload and share your model.
  – **URL:** Provide a link for your model.
- **BN tools:** List any specific tools, software, or applications that were used in the creation, analysis or presentation of the content.
- **Other comments:** Provide any additional information, context or comments that may be relevant to the BN, report, document or project. This is an open-text field where you can include any details that don’t fit in the other specified fields.

### Appendix A.2. Help Text for Structure Page

- **Structure (Default):** BNs have two fundamental aspects as follows: a structure (qualitative aspect) and parameters (quantitative aspect).
  The BN structure includes the definition of the model variables and the topological relationships between them. The structure of the BN is a Directed Acyclic Graph (DAG) where the variables are represented as nodes and the relationships as directed edges (arrows) between the nodes, indicating the direction of causal influence or probabilistic dependence.
  The BN parameters, covered in the next section, include the probabilistic details of the relationships, that is, the conditional probability tables or distributions, along with the values (states) variables can take and, for continuous variables, the range and discretisation.
- **Have you included a node dictionary?** A BN node (or variable) dictionary is a reference table that provides comprehensive information about the variables used in a BN model.
  The node dictionary serves as a valuable resource for understanding the variables and their meanings within the BN. By maintaining a comprehensive BN node dictionary, you ensure that stakeholders can easily comprehend and work with the model, fostering transparency and effective communication in decision making.
  Entries in the node dictionary will be relevant to both the structure and parameter aspects of the BN.
  *Key components of a node dictionary may include:*
  **Variable name:** This column lists the names of the variables in the BN. These names correspond to the random variables being modelled. Variable description: A brief description of each variable that explains its meaning, significance and relevance within the network.
  **Variable type:** This column specifies the variable type associated with each variable. It could be binary (e.g., True/False or 1/0), categorical (e.g., A, B, C), continuous (e.g., numeric values), or another variable type as appropriate.
  **States/Values:** For categorical or discrete variables, this section provides a list of possible values or states that the variable can take. For continuous variables, it may specify the range or distribution. Units of measurement: If applicable, this column indicates the units of measurement for each variable. For example, “Temperature (°C)” or “Income (USD$)”.
  **Parents/Dependencies:** This column lists the probabilistic dependencies of each variable, that is, its parents, along with a high-level description of the relationship to each, e.g., correlational, causal, barrier, inhibitor, requirement.
- **Where is the node dictionary (or alternative) reported?** Identify the location within the document where the node dictionary is defined. Specify the section or page number for easy reference.
- **Is the structure causal?** Causal BNs apply an explicitly causal interpretation to all, most or key selected arrows in the DAG.
  Causal BNs provide a structured way to represent how one variable influences or causes changes in another, making it an invaluable tool for decision making, prediction and understanding complex systems. BNs may be entirely causal, where all the arcs denote causality, or partially causal, where some arcs do not have a causal interpretation. In such cases, guidance must be given to users of the BN to make it clear which arcs are non-causal (or causal) to ensure the model can be used to support valid causal analyses.
  *Why use a causal BN?*
  A BN defines a joint probability distribution over a set of variables and makes no explicit claims about any causal relationships that may or may not hold between them. For a causal BN, the authors assert that some or all of the arrows represent causal relationships. If the authors make the claim that the arrows represent causal relationships, there is a much higher chance that the model can be used causally.
  Assuming that the arrows do represent genuine causal relationships, the model can be used for predicting or evaluating the outcomes of actions, decisions or interventions, something which non-causal BNs cannot do. Otherwise, the model can, at best, be used to estimate probabilistic associations.
- **Where is the structure defined?** Identify the location within the document where the structure is defined, e.g., a figure. Specify the section or page number for easy reference.
- **Select all applicable knowledge sources.** Indicate which knowledge sources were used to inform and build the probabilistic model represented by the BN, including any external information, data or expertise.
  **Problem owner(s):** This refers to individuals or entities with a direct stake in the problem being addressed by the BN model. Problem owners define the problem scope and objectives for the model, which will often include a requisite structure.
  **Analyst/Modeller(s):** These are the individuals responsible for the design and implementation of the model. They are banded alongside the modelling technique but not necessarily the problem domain. Analyst/modellers will often provide insights or expertise to improve model fidelity and simplicity.
  **Domain expert(s):** They often provide subject-specific knowledge that can significantly contribute to the accuracy and relevance of the BN model structure.
  **Literature:** This indicates whether the BN model structure incorporates information obtained from existing literature sources. This may include academic papers, books, or other published materials relevant to the model’s domain.
  **Data:** This indicates whether the BN model structure has been informed by data sources. Data-driven models rely on empirical evidence to establish relationships and patterns, making this knowledge source crucial for certain types of BNs.
- **Domain Expert(s) knowledge source selected:**
  – **Where is the elicitation process described?** Identify the location within the document where the structural elicitation process is detailed (this may be separate from any parameter elicitation). Specify the section or page number for easy reference.
  – **Which elicitation processes/protocols were used?** Describe the specific expert elicitation processes or protocols employed during the development of the BN.
    The elicitation protocols for the structure of the BN are not formalised or standardised, but the available methods for model building exist and their proponents recommend a participatory (and iterative) approach to model development that ends with a consensus model. The guidelines for eliciting the BN structure are informed by the same principles as the those used when eliciting the parameters (see next section).
  – **How many experts were involved?** Specify the number of experts who participated in the elicitation process.
    Provide details on the expert fields of expertise. Outline the fields of expertise covered by the participating experts. Include their qualifications and relevance to the subject matter. This may include academic backgrounds, professional experiences or specific areas of knowledge.
  – **Other comments:** Provide any additional comments or insights related to the expert elicitation process that may be pertinent for a comprehensive understanding.
- **Literature knowledge source selected:**
  – **Where are the literature sources described?** Identify the location within the document where the literature sources are detailed. Specify the section or page number for easy reference.
  – **Are literature sources cited in the node dictionary?** Indicate whether the literature sources are appropriately cited within the node dictionary or any other relevant documentation.
  – **Other comments:** Include any additional comments or observations related to the referenced literature that may contribute to a better understanding of the sources used.
- **Data knowledge source selected:**
  – **Where is the data learning process described?** Specify the section or page where the data learning process is described in the document. Offer a clear reference point for readers seeking information on the data learning methodology.
  – **What data learning tools/processes were used?** Describe the specific data learning tools/processes employed during the development of the BN. This may include machine learning algorithms, statistical methods or any other relevant techniques.
    A number of common tools are available for automatically selecting good structures for a BN, based on data, the goals and the existing expert knowledge available to the modelling group.
    For predictive models, common structures such as Naïve Bayes, minimum weighted spanning trees and Tree-Augmented Naïve Bayes often work well, and they are frequently available as options in common BN software packages.
    For both predictive and causal models, there is an even wider array of approaches, grouped under the heading of causal discovery. There are two main types of causal discovery algorithms as follows:
    **Constraint-based algorithms** that make use of statistical hypothesis tests to check for the presence or absence of direct causal relationships; and
    **metric-based algorithms** that search over the space of possible BNs to find high-scoring BNs, where the score is typically based on how well the BN predicts the dataset while penalising complex BNs.
    Where data are available, simple predictive approaches along with causal discovery approaches are always worth attempting, as they are at a minimum very good at identifying issues with the dataset and determining if the variable relationships align with expert expectations.
  – **Are data sources cited in the node dictionary?** Confirm whether the data sources utilized are appropriately cited within the node dictionary. This will help ensure transparency and traceability in data sourcing.
  – **Other comments:** Provide any additional comments or insights regarding the data learning process that may enhance the reader’s understanding or highlight noteworthy aspects.

### Appendix A.3. Help Text for the Parameters Page

- **Parameters (Default):** BNs have two fundamental aspects, a structure (qualitative) and a parameter (quantitative) aspect.
  The BN structure, covered in the previous section, includes the definition of the model variables and the topological relationships between them.
  The BN parameters include the probabilistic details of the relationships, that is, the conditional probability tables or distributions, along with the values (states) the variables can take and, for continuous variables, the range and discretisation.
- **Select the variable types used in the model.** Choose the appropriate variable types utilized in the BN model, which are either discrete, continuous, or a mixture of both.
- **Have you included state/discretisation definitions in the node dictionary?** Confirm whether the states or discretizations of variables have been included and properly documented in the node dictionary. This ensures clarity and reproducibility in understanding the variable characteristics.
  *Key components of a node dictionary may include the following:*
  **Variable name:** This column lists the names of the variables in the BN. These names correspond to the random variables being modelled. Variable description: A brief description of each variable that explains its meaning, significance and relevance within the network.
  **Variable type:** This column specifies the variable type associated with each variable. It could be binary (e.g., True/False or 1/0), categorical (e.g., A, B, C), continuous (e.g., numeric values) or another variable type as appropriate.
  **States/Values:** For categorical or discrete variables, this section provides a list of possible values or states that the variable can take. For continuous variables, it may specify the range or distribution. Units of measurement: If applicable, this column indicates the units of measurement for each variable. For example, “Temperature (°C)” or “Income (USD$)”.
  **Parents/Dependencies:** This column lists the probabilistic dependencies of each variable, that is, its parents, along with a high-level description of the relationship to each, e.g., correlational, causal, barrier, inhibitor, requirement.
- **Are the parameters described?** Indicate whether Conditional Probability Tables (CPTs), correlations, partial regression coefficients or equations associated with the BN model are available. Provide details on where this information can be found for reference.
- **Select all applicable knowledge sources:** Indicate which knowledge sources were used to inform and build the probabilistic model represented by the BN, including any external information, data or expertise.
  **Problem owner(s)**: This refers to individuals or entities with a direct stake in the problem being addressed by the BN model. Problem owners define the problem scope and objectives of the model, which will often include a requisite structure.
  **Analyst/Modeller(s):** These are the individuals responsible for the design and implementation of the model. They are banded alongside the modelling technique but not necessarily the problem domain. Analysts/modellers often provide insights or expertise to improve model fidelity and simplicity.
  **Domain expert(s):** They possess subject-specific knowledge that can significantly contribute to the accuracy and relevance of the BN model.
  **Literature:** Indicates whether the BN model incorporates information obtained from existing literature sources. This may include academic papers, books or other published materials relevant to the model’s domain.
  **Data:** Indicates whether the BN model has been informed by data sources. Data-driven models rely on empirical evidence to establish relationships and patterns, making this knowledge source crucial for certain types of BNs.
- **Domain expert(s) knowledge source selected:**
  – **Where is the elicitation process described?** Identify the location within the document where the elicitation process is detailed. Specify the section or page number for easy reference.
  – **Which elicitation processes/protocols were used?** Describe the specific expert elicitation processes or protocols employed during the development of the BN.
    The recommended expert elicitation protocols for eliciting parameters are those which are structured. Building on the community understanding of what a structured expert elicitation protocol may mean, where quantitative elicitation is concerned, the following elements can be identified as crucial:
    1. Asking questions that have clear operational meanings.
    2. Asking at least four to six diverse experts.
    3. Following transparent methodological rules, such that the process is traceable and repeatable.
    4. Mitigating cognitive (individual and group) biases.
    5. Providing opportunities for evaluation and validation.
    The main differences between the most established protocols are the level of interaction they allow between experts and the way final aggregation of judgements is treated (to represent the group judgement).
    Three of the most established protocols for structured expert judgement are as follows:
    **IDEA protocol:** A Delphi-like protocol that uses two rounds of estimation, feedback and discussion and mathematical aggregation of judgements.
    **SHELF protocol:** A behavioural aggregation protocol which allows for extensive discussion and strives for consensus judgements.
    **Cooke’s protocol:** A protocol that allows for minimal interaction, using mathematical aggregation and always using calibration questions for validation purposes.
  **How many experts were involved?** Specify the number of experts who participated in the elicitation process.
  **Provide details on the experts’ fields of expertise.** Outline the fields of expertise covered by the participating experts. Include their qualifications and relevance to the subject matter. This may include academic backgrounds, professional experiences or specific areas of knowledge.
  **Other comments:** Provide any additional comments or insights related to the expert elicitation process that may be pertinent for a comprehensive understanding.
- **Literature knowledge source selected:**
  – **Where are the literature sources described?** Identify the location within the document where the literature sources are detailed. Specify the section or page number for easy reference.
  – **Are literature sources cited in the node dictionary?** Indicate whether the literature sources are appropriately cited within the node dictionary or any other relevant documentation.
  – **Other comments:** Include any additional comments or observations related to the literature details that may contribute to a better understanding of the sources used.
- **Data knowledge source selected:**
  – **Where is the data learning process described?** Specify the section or page where the data learning process is described in the document. Offer a clear reference point for readers seeking information on the data learning methodology.
  – **What data learning tools/processes were used?** Describe the specific data learning tools/processes employed during the development of the BN. This may include machine learning algorithms, statistical methods or any other relevant techniques.
    Data learning processes differ depending on whether the BN model is discrete or continuous. For discrete BNs, there are two key methods for performing parameterisation.
    **Count learning:** When data are complete (there are no missing values and no hidden variables), this is the primary approach for performing data learning. It is simple and fast, and it corresponds to the maximum likelihood estimate of the parameters.
    **EM learning:** EM (Expectation-Maximisation) is a method that has been proven to work well with missing data. It is an iterative method that estimates the parameters based on the complete data; it then uses the completed data to re-estimate the parameters, then back and forth between updating the parameters and updating the data. While EM is guaranteed to converge, it is not guaranteed to converge to the same thing with each run; hence, multiple EM runs are advised, particularly when there is a large amount of missing data.
    Other methods exist as well, such as gradient descent, that can work better or more efficiently than EM learning, depending on the circumstances. For continuous BNs, there is an array of methods that often depend on the functional form of the nodes. Parameterisation is most commonly done with logit and other regression approaches, as well as rank correlations and kernel density estimators.
  – **Were priors used? If yes, provide details.** In BNs, parameter learning involves estimating the probabilities or parameters associated with the conditional probability distributions (CPDs) of each node given its parents. Priors play a crucial role in Bayesian parameter learning by incorporating existing knowledge or beliefs about the parameters before observing any data. If you used priors, please provide details on their source and identify the location within the document where they are detailed.
  – **Are data sources cited in the node dictionary?** Confirm whether the data sources utilized are appropriately cited within the node dictionary. This will help ensure transparency and traceability in data sourcing.
  – **Other comments** Provide any additional comments or insights regarding the data learning process that may enhance the reader’s understanding or highlight noteworthy aspects.

### Appendix A.4. Help Text for Evaluation Page

- **Evaluation (Default)** BN evaluation involves assessing the performance, reliability and correctness of a BN model. This process ensures that the model accurately represents the underlying system or phenomenon it is intended to capture.
- **What validation/verification processes were used?** Describe the specific validation and verification processes employed to assess the performance and reliability of the model. This may include cross-validation, holdout validation or other relevant techniques.
  BN validation involves assessing the degree to which the model’s predictions align with observed data or empirical evidence. It entails comparing the BN’s probabilistic predictions or inferences with real-world observations to ascertain the model’s accuracy, reliability and generalizability.
  BN verification refers to confirming that the implemented model accurately reflects the specified structure and conditional probability distributions outlined in the model’s graphical representation or mathematical formulation. It involves verifying that the BN software or framework correctly implements the intended model without coding errors or inconsistencies.
- **What sensitivity analysis processes were used?** Outline the sensitivity analysis methods utilized to evaluate the model’s responsiveness to changes in input parameters. This ensures a comprehensive understanding of the model’s behaviour under varying conditions.
  BN sensitivity analysis is a systematic investigation conducted to evaluate how variations or uncertainties in the input variables of a BN model affect its outputs or predictions. It assesses the robustness and reliability of the model’s inferences by examining the sensitivity of the results to changes in the parameters, evidence or structure of the network.
- **Where is the evaluation reported?** Identify the location within the document where the BN evaluation is detailed. Specify the section or page number for easy reference.
- **Select all applicable knowledge sources.** Indicate which evaluation sources were used to validate and verify the probabilistic model represented by the BN, including any external information, data or expertise.
  **Problem owner(s):** This refers to individuals or entities with a direct stake in the problem being addressed by the BN model.
  **Analyst/Modeller(s):** These are the individuals responsible for the design and implementation of the model. They are banded alongside the modelling technique but not necessarily the problem domain.
  **Domain expert(s):** They possess subject-specific knowledge that can significantly contribute to the accuracy and relevance of the BN model.
  **Literature:** Select this option if the BN model evaluation incorporates information obtained from existing literature sources. This may include academic papers, books or other published materials relevant to the model’s domain.
  **Data:** Choose this if the BN model has been evaluated against data sources.
- **Domain expert(s) knowledge source selected:**
  – **Where is the expert validation reported?** Identify the location within the document where the expert validation processes are detailed. Specify the section or page number for easy reference.
  – **How were experts used?** Describe the way experts were involved in the evaluation process. This could include expert interviews, validation workshops or other methods used to incorporate expert knowledge.
  – **Were the experts different from model development?** Specify whether the experts involved in the evaluation phase were distinct from those engaged in the initial development of the model. This helps in assessing independent perspectives and validation.
  – **How many experts were involved?** Specify the number of experts who participated in the evaluation process.
  – **Provide details on the expert fields of expertise.** Outline the fields of expertise covered by the participating experts. Include their qualifications and relevance to the subject matter. This may include academic backgrounds, professional experiences or specific areas of knowledge.
  – **Other comments:** Provide any additional comments or insights regarding the expert evaluation process that may enhance the reader’s understanding or highlight noteworthy aspects.
- **Data knowledge source selected:**
  – **Where is the data validation reported?** Identify the location within the document where the data validation processes are detailed. Specify the section or page number for easy reference.
  – **How were data used?** Describe the role of data in the evaluation process. This could involve using new data sets, additional observations or specific methodologies to assess the model’s performance.
  – **Were the data different from model development?** Specify whether the data used in the evaluation phase differed from that employed in the initial model development. This information aids in understanding the generalizability and robustness of the model.
  – **Are data sources cited in node dictionary?** Confirm whether the sources of data used in the evaluation are properly cited. Proper citation ensures transparency and allows for the reproducibility of the evaluation process.
  – **Other comments:** Provide any additional comments or insights regarding the data evaluation process that may enhance the reader’s understanding or highlight noteworthy aspects.
